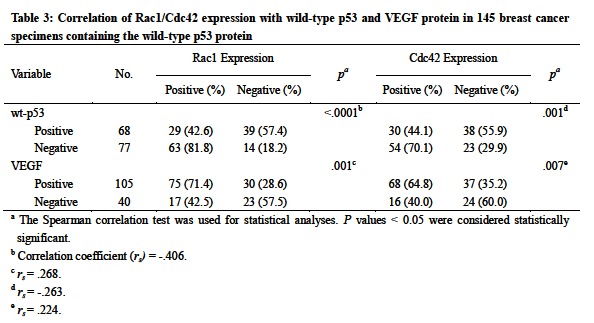# Correction: Role of Activated Rac1/Cdc42 in Mediating Endothelial Cell Proliferation and Tumor Angiogenesis in Breast Cancer

**DOI:** 10.1371/annotation/0f309de5-f22e-4f59-b3a2-5991e1f3ce28

**Published:** 2013-06-10

**Authors:** Ji Ma, Yan Xue, Wenchao Liu, Caixia Yue, Feng Bi, Junqing Xu, Jian Zhang, Yan Li, Cuiping Zhong, Yan Chen

There were errors in Tables 1 and 3. Correct version of the tables are available here:

Table 1: 

**Figure:**
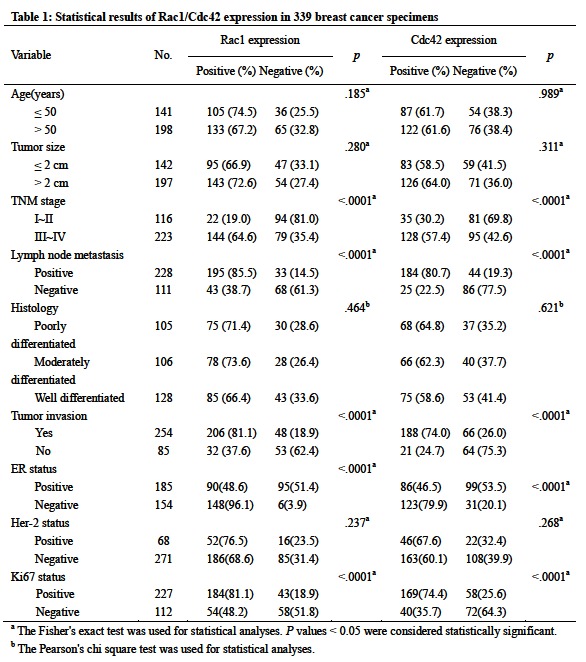


Table 3: 

**Figure:**